# *Streptococcus equi* subsp. *zooepidemicus* isolates from equine infectious endometritis belong to a distinct genetic group

**DOI:** 10.1186/1297-9716-44-26

**Published:** 2013-04-18

**Authors:** Camilla Dooleweerdt Rasmussen, Maria Mathilde Haugaard, Morten Roenn Petersen, Jesper Møller Nielsen, Hanne Gervi Pedersen, Anders Miki Bojesen

**Affiliations:** 1 Department of Large Animal Sciences, Section of Veterinary Reproduction and Obstetrics, Faculty of Health and Medical Sciences, University of Copenhagen, Dyrlaegevej 68, Frederiksberg C DK-1870, Denmark; 2 Department of Large Animal Sciences, Section of Medicine and Surgery, Faculty of Health and Medical Sciences, University of Copenhagen, Hoejbakkegaard Allé 5, Taastrup DK-2630, Denmark; 3 Fertility Clinic, Rigshospitalet, Section 4071, University Hospital of Copenhagen, Blegdamsvej 9, Copenhagen DK-2100, Denmark; 4 Ansager Dyrehospital (Ansager Large Animal Hospital), Gartnerhaven 5, Ansager DK-6823, Denmark; 5 Department of Disease Biology, Faculty of Health and Medical Sciences, University of Copenhagen, Stigboejlen 4, Frederiksberg C DK-1870, Denmark

## Abstract

*Streptococcus equi* subsp. *zooepidemicus* is the pathogen most commonly isolated from the uterus of mares. *S. zooepidemicus* is an opportunistic pathogen and part of the resident flora in the caudal reproductive tract. The aim of this study was to investigate whether a genotypically distinct subpopulation of *S. zooepidemicus* is associated with endometritis in the mare, by genotyping and comparing uterine *S. zooepidemicus* strains with isolates from the vagina and clitoral fossa. Mares with (*n* = 18) or without (*n* = 11) clinical symptoms of endometritis were included. Uterine samples were obtained using a guarded endometrial biopsy punch, whereas a swab was used to recover samples from the cranial vagina and the clitoral fossa. If *S. zooepidemicus* was present, up to three colonies were selected from each anatomical location (max. 9 isolates per mare). Bacterial isolates were characterized by pulsed-field gel electrophoresis (PFGE) and multilocus sequence typing (MLST). *S. zooepidemicus* was isolated from the endometrium of 12 mares. A total of 88 isolates were analyzed by PFGE: 31 from the endometrium, 26 from the cranial vagina and 31 isolates from the clitoral fossa. For MLST 21 isolates were chosen. Results demonstrated a higher genetic similarity of the isolates obtained from infectious endometritis compared to isolates obtained from the caudal reproductive tract. In conclusion, we demonstrate for the first time that a genetically distinct group of *S. zooepidemicus* is associated with infectious endometritis in the mare.

## Introduction

*Streptococcus equi* subspecies *zooepidemicus* (*S. zooepidemicus*) is a beta-hemolytic Gram-positive Lancefield group C bacterium found in a wide range of species including horses, pigs, cattle, goats, poultry, dogs and humans [1]. It appears to be part of the normal bacterial microflora of the upper respiratory tract and caudal reproductive tract of horses, and is also found in healthy carriers of other species such as pigs and monkeys [2,3]. *S. zooepidemicus* is an opportunistic pathogen associated with a wide variety of diseases e.g. pneumonia, septicemia, mastitis, placentitis and endometritis [1]. In the pathogenesis of a respiratory infection with *S. zooepidemicus,* factors associated with the bacteria, as well as with the host, appear to play a major role in the clinical appearance and outcome of the disease [2,4].

*S. zooepidemicus* is the most frequently isolated pathogen from the uterus of the mare [5]. The prevailing hypothesis is that isolates of *S. zooepidemicus,* residing in the lower reproductive tract, cause infectious endometritis by an ascending infection in a random manner primarily governed by the uterine defense mechanisms of the mare [6-8]. The clitoral fossa, clitoral sinuses and the vagina have been suggested as possible bacterial reservoirs [5,9]. For *S. zooepidemicus* to be able to reach the uterus it has to pass three physio-anatomical barriers; the vulva, the vestibulovaginal sphincter and the cervix. Poor anatomical conformation of the internal and external reproductive organs may impair these barriers and allow bacteria to ascend into the uterus [10]. Contamination of the uterus also takes place during live cover, artificial insemination, or iatrogenically [11]. Whether or not *S. zooepidemicus* will establish an uterine infection has been described to depend primarily on factors related to the uterine defense mechanisms more so than to factors associated with the bacteria alone [11,12]. Virulence factors such as fibronectin-binding proteins [13], hyaluronic capsule [14], M-like proteins [15] and Fc receptors [16] have been identified for *S. zooepidemicus* yet no studies have to our knowledge demonstrated the significance of these factors at establishment of endometritis in mares. To address why *S. zooepidemicus* continues to be so successful at causing endometritis, in spite of the increased knowledge of the pathogenesis and more efficient methods of treatment, we aimed to assess whether specific strains of *S. zooepidemicus* are better adapted to the uterine environment than others.

Specifically, the aim of this study was to investigate whether a genotypically distinct subpopulation of *S. zooepidemicus* is associated with endometritis in the mare, by genotyping and comparing uterine *S. zooepidemicus* strains with isolates from the vagina and clitoral fossa, using pulsed-field gel electrophoresis (PFGE) and multilocus sequence typing (MLST).

## Materials and methods

### Equipment and sample collection

The mares (*n* = 29) included in the study were of different breeds and aged 3 to 25 years. All the mares originated from individual herds. Sample collection took place on a stallion stud farm. Mares with (*n* = 18) or without (*n* = 11) clinical symptoms of endometritis, such as intrauterine fluid, were included. From each mare a sample was taken from three anatomical locations: the uterus, the cranial part of the vagina and the clitoral fossa. The samples were obtained with no regards to stage of cycle; hence both estrus and diestrus samples are represented in the study. All samples were collected between April and June in the 2007 breeding season.

The mares were restrained in an examination stock and the tail was wrapped. At first samples from the clitoral fossa were collected using a sterile swab (BBL™CultureSwab™ with Stuart liquid medium, BD, Franklin Lakes, NJ, USA), without touching the vulva lips. If the clitoral fossa was too dry for proper sampling, the sterile swab was lubricated with either sterile saline or transport medium (Stuart liquid medium) before sampling. Before collecting samples from the cranial vagina and the uterus, the perineal area was washed with warm water and dried with paper towels. The operators hand and arm was covered with a sterile rectal sleeve. A double-guarded swab (Equi-Vet®; Kruuse, Marslev, Denmark) was guided manually to the cranial part of the vagina. The swab was allowed contact with the vaginal surface for at least 30 s, while being rotated. Uterine samples were collected using a guarded endometrial biopsy punch (Equi-vet®, Kruuse, Marslev, Denmark) technique, as described by Nielsen [17]. A sterile stainless steel speculum (Equi-vet®, Kruuse) was guided through the vagina and inserted into the cervix. The biopsy instrument was then inserted into the speculum and passed into the uterus for sample collection. After sample collection the biopsy instrument was retracted into the speculum and both the instruments were removed from the mare. Using the sterile steel speculum, the risk of contaminating the endometrial biopsy with bacteria from the caudal reproductive tract, is markedly reduced.

### Microbiology

Samples were smeared onto a blood agar plate (5% calf blood added to blood agar base, Oxoid, Roskilde, Denmark) within 8 h of collection and incubated at 37°C for 24 h in atmospheric air. Colonies were identified as *S. zooepidemicus* based on their colony morphology, microscopic appearance, Gram-positive appearance and their ability to ferment lactose, sorbitol and ribose but not trehalose. From the mixed cultures of the clitoral fossa only *S. zooepidemicus* was isolated for analysis. Only isolates originating from pure cultures (≥ 90% of all colonies) of the uterine and vaginal samples were included in the study. If *S. zooepidemicus* was present; up to 3 colonies were selected from each anatomical location i.e. maximum 9 isolates per mare. Isolates were stored at −80°C in 15% glycerol until further analysis.

### Pulsed-field gel electrophoresis (PFGE)

To prepare the bacterial DNA for PFGE each isolate was incubated in brain heart infusion broth (Oxoid, Roskilde, Denmark) overnight at 37°C. The culture was washed three times and adjusted to 10^10^ CFU/mL in NaCl-EDTA buffer (75 mM NaCl/25 mM EDTA, pH 7.4). From the bacterial suspension agarose blocks were casted (10 × 6 × 2 mm) (Certified™ Megabase Agarose, Bio-Rad®, Hercules, CA, USA). The agarose blocks were incubated overnight at 37°C in EC lysis buffer (6 mM Tris/1 M NaCl/100 mM EDTA/0.5% Brij-58/0.2% Sodium deoxycholate/0.5% N-lauroylsarcosine, pH 8.0) containing 20 μL Mutanolysin (Sigma-Aldrich®, Broendby, Denmark). The agarose blocks were then washed in Tris-EDTA buffer (10 mM Tris/10 mM EDTA, pH 7.4) and incubated overnight at 56°C in ES buffer (1% N-lauroylsarcosine/0.5 M EDTA, pH 9.5) containing Proteinase K (1 mg/mL) (Roche Diagnostics^©^, Hvidovre, Denmark). After incubation the agarose blocks were washed in Tris-EDTA buffer twice and cut into 4 pieces about 2 mm wide. The smaller agarose blocks were transferred to an Eppendorf tube containing the *Sma*I restriction enzyme (New England Biolabs Inc.®, Ipswich, MA, USA), the accompanying enzyme buffer (NEBuffer 4 (B7004S), New England Biolabs Inc.®) and bovine serum albumin (B9001S, New England Biolabs Inc.®). The agarose blocks were incubated overnight at 25°C. To stop the enzymatic reaction the enzyme solution was removed and Tris-EDTA buffer was added. The agarose blocks from the different isolates were then casted in a GTG agarose gel (SeaKem®GTG®Agarose, Lonza, Rockland, ME, USA) and the electrophoresis was set to run for 20 h at 5.6 volts/cm and at a 120° included angle (CHEF-DR® III, Bio-Rad, CA, USA) The Midrange II PFG marker (New England Biolabs Inc.®, Ipswich, MA, USA) was used. The gel was stained with ethidium bromide for 20 min. and photographed. All chemicals used in the buffers were purchased at Sigma-Aldrich (Sigma-Aldrich®, Broendby, Denmark).

### Multilocus sequence typing (MLST)

An isolate representing each major PFGE genotype was characterized by MLST. Each isolate was incubated in brain heart infusion broth overnight at 37°C. DNA was then purified using DNeasy® Blood and Tissue kit according to the manufacturer’s instructions (Qiagen, Denmark). PCR amplification of seven housekeeping genes carbamate kinase (*arcC*), ribonucleoside-diphosphate reductase (*nrdE*), propyl-tRNA synthetase (*proS*), signal peptidase I (*spi*), thymidylate kinase (*tdk*), triosephosphate isomerase (*tpi*) and acetyl-CoA acetyltransferase (*yqiL*) was carried out as described by Webb et al. [18]. Sequencing was performed by Macrogen® (Seoul, Korea).

### Analysis of genetic relatedness

The PFGE banding patterns were analyzed for genetic relatedness using GelCompar® (Applied Maths, Sint-Martens-Latem, Belgium). The Unweighted Pair Group Method using Aritmetic Averages (UPGMA) and the Pearson’s similarity coefficient expressed as percentage was used to analyze the genetic relatedness of the *S. zooepidemicus* isolates from the PFGE analysis. Bacterial isolates with a Pearson similarity coefficient of > 90% was considered clonal.

The MLST sequence data from Macrogen® (Seoul, Korea) was assembled and analyzed using the MLST module in CLC Main Workbench 6.0 (CLC Bio®, Aarhus, Denmark) and the *S. zooepidemicus* MLST scheme described by Webb et al. [18]. The MLST results were uploaded to the MLST database [19] and a population snapshot of the *S. zooepidemicus* group was made using eBURST v3 [20,21]. The stringent group definition (sharing 6 of 7 loci) for clonal complexes was used. The MLST results was analyzed further using ClonalFrame v1.1 [22,23]. In total three independent runs were performed, each consisting of 300 000 iterations. The first 150 000 iterations (burn-in) in each run were discarded to allow for convergence, followed by 150 000 iterations of which every 100^th^ generation was sampled (thinning interval). Thus, each run produced a sample size of 1501 of the posterior. Convergence of the three independent runs was checked by the Gelman and Rubin statistics included in the ClonalFrame GUI [24]. The posterior samples of the three independent runs were combined resulting in a sample size of 4503, and a majority-rules consensus tree was made. MEGA4 [25] was used to make a graph of the majority-rules consensus tree from ClonalFrame.

Fisher’s exact test was used to evaluate the difference in the distribution of *S. zooepidemicus* isolates from the endometrium in the genetic clusters.

## Results

### Microbiology

*S. zooepidemicus* was isolated from the endometrium of 12 out of the 18 mares (66.7%) with clinical symptoms of endometritis. *S. zooepidemicus* was not isolated from the endometrium of mares with no clinical symptoms of endometritis (0/11 mares). In total 88 *S. zooepidemicus* isolates were collected: 31 isolates from the endometrium of 12 mares, 26 from the cranial vagina of 12 mares, and 31 from the clitoral fossa of 14 mares. Originally 36 endometrial isolates were recovered from the 12 mares. However, only 31 of 36 of the endometrial isolates could be retrieved from storage.

### PFGE

**Figure 1:**
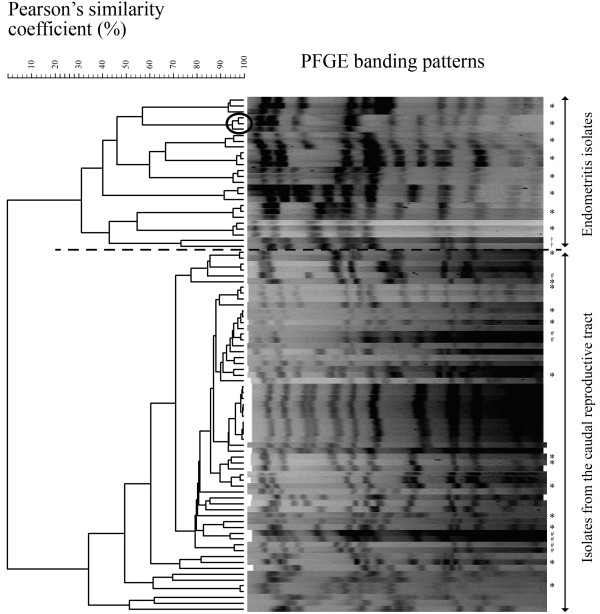
**Genetic relatedness of the *S. zooepidemicus *isolates analyzed by PFGE.** Genetic relatedness between the individual banding patters from the PFGE of the 88 *S. zooepidemicus* isolates. The isolates cluster in two groups based on the origin of the sample. Circle marks three clonal isolates from the uterus of one mare. (*) *S. zooepidemicus* isolates chosen for MLST. (#) *S. zooepidemicus* isolates from endometritis in the cluster of isolates from the caudal reproductive tract. (†) *S. zooepidemicus* isolates from the caudal reproductive tract in the cluster of endometritis isolates.

The genetic relatedness between the individual banding patterns of the *S. zooepidemicus* isolates from the PFGE is shown in Figure 1. The figure shows that the *S. zooepidemicus* isolates cluster in two major groups with very low genetic relatedness (Pearson similarity < 10%). The upper group comprised 26 isolates and the lower group 62 isolates. Of the 26 isolates in the upper group, 24 isolates (92.3%) originated from the endometrium. The remaining two isolates were isolated from the cranial vagina of a mare, which also had *S. zooepidemicus* isolated from the endometrium. Both vaginal isolates were genetically closer related to the uterine isolates from the same mare, than to uterine isolates from other mares. In the lower group only 7 out of the 62 isolates came from the endometrium (11.3%), while the remaining 55 isolates had been isolated from the cranial vagina or the clitoral fossa. The proportion of *S. zooepidemicus* isolates from the endometrium in the two genetic clusters was significantly different (*p* < 0.0001).

When comparing the genetic relatedness of the isolates taken from the same location in the same mare two observations were made. First, the uterine isolates showed a very high genetic relatedness within each individual mare, indicating that only one genotype appeared to be present in the uterine compartment. This is shown in the dendrogram by the grouping of three isolates per mare in the endometritis cluster (Figure 1). Secondly, the genetic diversity among the *S. zooepidemicus* isolates appeared to increase the further caudal in the reproductive tract they originated. In mares where more than one *S. zooepidemicus* isolate was collected, 4 out of 8 (50%) had more than one clone in the cranial vagina, and 6 out of 9 (67%) had more than one clone at the clitoral fossa.

### MLST

MLST was used as a second method to analyze the genetic relatedness of the *S. zooepidemicus* isolates. Of the 88 *S. zooepidemicus* isolates analyzed by PFGE, 21 were chosen for MLST analysis: 8 endometritis isolates, 6 from the cranial vagina, and 7 from the clitoral fossa. The isolates were chosen to account for the entire genetic diversity revealed by PFGE, and none of the isolates originated from the same mare (Figure 1).

**Figure 2:**
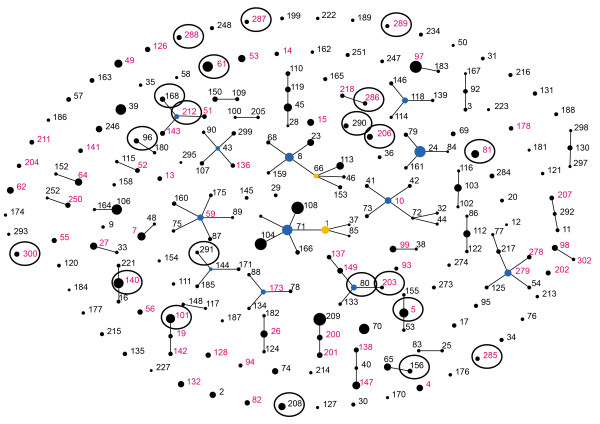
**eBURST population snapshot of equine *S. zooepidemicus.*** Population snapshot of equine *S. zooepidemicus* STs from the MLST database using eBURST. STs which has been isolated from abortion or uterine disease are written in pink. The 21 ST from this study is marked with a black circle. Primary founders are blue and subgroup founders are yellow. The area of the circle indicates the prevalence of the ST in the database. Clusters of linked STs correspond to clonal complexes.

A total of 21 different allelic profiles or sequence types (ST) were identified. Thirteen had a ST already registered in the MLST database, whereas eight were new to the database (Table 1). To illustrate the relationship between the 21 STs obtained from this study and the existing equine *S. zooepidemicus* STs in the MLST database an eBURST population snapshot was made (Figure 2). In total 11 of the 21 STs from this study clustered in clonal complexes (CCs) in the eBURST population snapshot of all equine *S. zooepidemicus* registered in the MLST database. ST-80 and ST-203 from this study represent vaginal isolates and in the eBURST they form a CC with other equine *S. zooepidemicus* STs isolated from the reproductive organs. Similarly, ST-168 (clitoral) and ST-212 (vaginal) also clustered in a CC with other equine *S. zooepidemicus* STs isolated from the reproductive organs (Figure 2). To investigate the genetic relatedness of the 21 STs from the present study, ClonalFrame was used. ClonalFrame uses a Monte-Carlo Markov chain algorithm that infers clonal relations while taking into account the effect of recombination [22]. The resulting majority-rules consensus tree showed that 6 of the 8 endometritis isolates and three isolates from the caudal reproductive tract clustered together creating a subcluster when compared to the remaining isolates. The three isolates from the caudal reproductive tract in the endometritis cluster represented two isolates from the clitoral fossa and one vaginal isolate. In the PFGE analysis they represent different subgroups of the lower cluster of isolates from the caudal reproductive tract. *S. zooepidemicus* isolates from endometritis clustered significantly together in the endometritis subcluster, compared to the remaining cluster primarily comprised of isolates from the caudal reproductive tract (*p* = 0.0318).

**Table 1:** **Sequence type (ST) and allelic profile of the 21** ***S. zooepidemicus *isolates analyzed by MLST**

**ST**	**Origin**	***arcC***	***nrdE***	***proS***	***spi***	***tdk***	***tpi***	***ygiL***
5	*Endometritis*	5	3	3	5	3	5	5
81	*Endometritis*	9	5	9	4	1	9	1
140	*Endometritis*	3	3	10	22	10	5	12
285*	*Endometritis*	1	3	1	2	1	3	60
286*	*Endometritis*	8	3	10	6	30	3	22
287*	*Endometritis*	9	3	10	4	18	19	40
288*	*Endometritis*	2	49	1	54	1	1	61
289*	*Endometritis*	38	11	10	7	1	34	6
80	*Vagina*	2	4	4	7	4	5	20
101	*Vagina*	12	12	4	45	11	5	34
156	*Vagina*	4	3	6	2	1	19	26
203	*Vagina*	2	4	4	7	4	5	43
212	*Vagina*	10	14	22	14	15	10	33
290*	*Vagina*	2	10	9	55	13	23	16
61	*Clitoris*	18	11	20	6	17	16	3
96	*Clitoris*	2	6	14	4	4	14	16
168	*Clitoris*	10	14	1	14	15	10	33
206	*Clitoris*	2	10	9	6	5	10	16
208	*Clitoris*	8	18	1	24	1	5	49
291*	*Clitoris*	21	2	10	2	1	13	10
300*	*Clitoris*	27	3	4	45	1	5	6

*ST new to the database.

## Discussion

The results from this study indicate that *S. zooepidemicus* strains associated endometritis in mares belong to a genetically distinct subpopulation. This adds a new perspective to the pathogenesis of equine infectious endometritis, as infectious endometritis may not just be caused by a random contamination of *S. zooepidemicus* from the caudal reproductive tract, but is likely to be caused by more specialized endometrial pathogenic strains. This indicates that it is not only the efficiency of the uterine defense mechanisms that determines whether *S. zooepidemicus* will establish an infection, but rather the interaction between the *S. zooepidemicus* strain and compromised uterine defense mechanisms.

**Figure 3:**
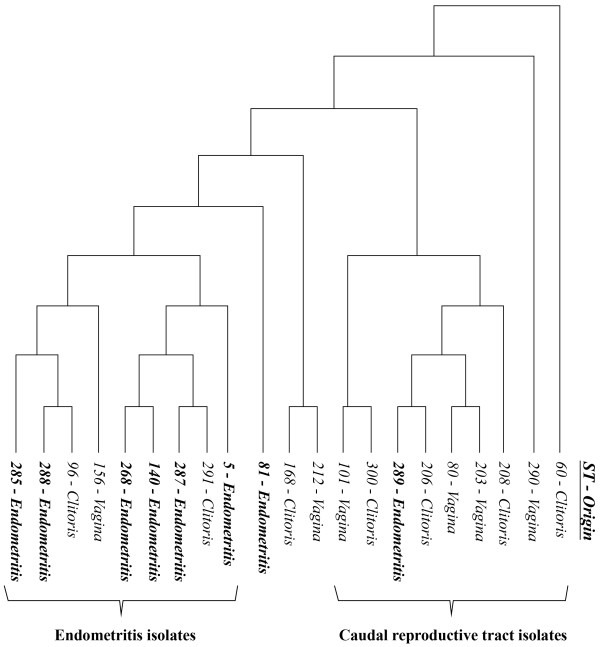
**Genetic relatedness of the *S. zooepidemicus *isolates analyzed by ClonalFrame.** A majority-rules consensus tree made from the posterior sample of the 3 independent runs in ClonalFrame of the MLST results from the 21 *S. zooepidemicus* isolates. Isolates from endometritis cluster together compared to isolates from the caudal reproductive tract (*p* = 0.0318).

The *S. zooepidemicus* population is genetically very diverse as illustrated by the eBURST population snapshot (Figure 2). A few studies have investigated whether specific strains are associated with specific diseases and characterized the genetic diversity and epidemiology of *S. zooepidemicus*[2,18,26]. Webb et al. [18] indicated that strains isolated from abortion and uterine disease clustered non-randomly when analyzed by MLST and compared to strains from other diseases e.g. respiratory disease or wound infection, yet too few strains were analyzed to permit statistically solid conclusions. The aim of the present study was to genotype and compare multiple *S. zooepidemicus* isolates originating from the entire reproductive tract of individual mares, in order to investigate if a specific subpopulation of *S. zooepidemicus* was associated with endometritis. The results from both PFGE (Figure 1) and the ClonalFrame analyze (Figure 3) suggests that *S. zooepidemicus* isolates from infectious endometritis are genetically different from isolates collected from the caudal reproductive tract (cranial vagina and the clitoral fossa). There is still some genetic diversity between endometritis isolates from different mares, which indicates that no single clone of *S. zooepidemicus* but rather a subgroup of related clones causes endometritis in mares. Luque et al. also studied the genetic relatedness of *S. zooepidemicus* isolates from equine infectious endometritis using PFGE, and they found a low genetic relatedness among the endometritis isolates [27]. They did not compare the genetic relatedness of the endometritis isolates with other *S. zooepidemicus* isolates e.g. from the caudal reproductive tract of the same mares. It is therefore, from this study, not clear whether endometritis isolates would have clustered, in spite of their genetic differences, if they had been compared with isolates from other sources.

In the results from our study two vaginal *S. zooepidemicus* strains clustered with the 24 endometrial strains in the upper endometritis cluster in the PFGE analysis (Figure 1). The two vaginal strains were closer related to the endometrial strains from the same mare than to the endometrial strains from other mares. This finding is to be expected since bacteria and debris is expelled caudally by the uterine contractions through the cervix as a part of the uterine clearance mechanisms [6]. The strains collected at the cranial part of the vagina could therefore be strains originating from the uterus, hence the high genetic relatedness with the endometrial strains. The opposite finding, that a few endometrial strains clustered with strains from the caudal reproductive tract (Figure 1), may illustrate the pathogenesis of an ascending infectious endometritis, in that bacteria are carried from the caudal reproductive tract to the uterus where the endometrial pathogenic strains survive and establish an infection.

The concept of specific endometrial pathogenic *E. coli* strains has been demonstrated in cattle by Sheldon et al. [28]. The endometrial pathogenic *E. coli* was more adherent and invasive to endometrial cells than strains from unaffected animals, but they lacked the typically pathogenicity genes associated with virulence. With regards to *Streptococcus agalactiae* Fluegge et al. demonstrated that invasive human neonatal strains represented only a small proportion of the entire population [29]. The observed genetic difference between the endometritis strains and strains from the caudal reproductive tract found in this study may be due to a difference in the virulence genes of *S. zooepidemicus.* Acquisition of specific virulence factors could enhance the pathogenicity of some strains in the uterus. As mentioned in the introduction *S. zooepidemicus* may encode several virulence factors that can alter the interaction between the bacteria and the immune response of the host e.g. fibronectin-binding proteins, hyaluronic capsule, M-like proteins and Fc receptors [13-16]. Petersen et al. showed that in chronically infected mares *S. zooepidemicus* resides deep in the endometrium [30]. This could perhaps be due to the expression of specific virulence genes that facilitates colonization and evasion of the host immune response in order for the bacteria to survive and establish a chronic infection. Currently no virulence factors associated with *S. zooepidemicus* originating from the reproductive tract have been identified and to our knowledge none of the putative virulence genes in *S. zooepidemicus* have been confirmed in pathogenicity studies aiming at comparing wildtype and mutant strains in horses. As the vast majority of *S. zooepidemicus* are moderately pathogenic bacteria, differences in their pathogenic potential are likely to depend on variations in virulence factor expression levels. Thus although we provide solid support for existence of a genetic subpopulation of endometritis associated *S. zooepidemicus*, we find that there is a very long way to go before the complete picture delineating individual factors governing the bacteria host interactions between *S. zooepidemicus* and mares is established. Future studies will therefore investigate the virulence genes of the *S. zooepidemicus* strains from this study to determine if certain virulence genes are associated with the endometritis strains.

Our results also showed that endometritis in the individual mare is caused by a single genotype rather than by a mixed group of strains. Similar findings were reported by Kuroiwa et al. who demonstrated that, when analyzing the *szp* genotype of 5 colonies from the same uterine sample, 9 out of 10 mares had only one clone represented [31]. This is in contrast to the findings of Luque et al. who showed that *S. zooepidemicus* isolated from the endometrium of the same mare showed low genetic relatedness [27]. One reason for this result could be that they only compared one isolate per sampling event taken a month apart. It was therefore not possible to compare the genetic relatedness of multiple isolates taken from the same sample. This made the qualitative assessment difficult, since the mare in the meantime could have been re-infected with another strain.

Unlike the monoculture of the *S. zooepidemicus* found in the uterus in each of the mares, the genetic diversity of the *S. zooepidemicus* amongst the collected isolates in this study was found to increase in samples collected most caudal in the reproductive tract. The increase in genetic diversity from the vagina to the clitoral fossa most likely reflects the increasing degree of environmental contamination with commensal *S. zooepidemicus* the closer to the external orifice the sample is taken [9].

*S. zooepidemicus* is an opportunistic pathogen that through evolution successfully has adapted to many different host species and tissues. To our knowledge this is the first study to demonstrate that specific *S. zooepidemicus* strains may have evolved to become better adapted to the equine uterine environment compared to other strains. This could explain why this bacterium continues to be so successful in causing endometritis in the mare despite the many and progressive attempts to treat and eliminate it.

In conclusion the results from this study showed that *S. zooepidemicus* isolated from mares with infectious endometritis had a higher genetic similarity than isolates collected from the caudal reproductive tract within the same mare and between mares. This indicates that a genetically distinct subpopulation of *S. zooepidemicus* is associated with infectious endometritis in the mare, and that some strains are better suited to survive in and colonize the endometrium. Future research will focus on identifying the specific factors characterizing these bacterial strains.

## Abbreviations

PFGE: Pulsed-field gel electrophoresis; S. zooepidemicus: *Streptococcus equi* subsp. *zooepidemicus*; CC: Clonal complex; ST: Sequence type; MLST: Multilocus sequence typing.

## Competing interests

The authors declare that they have no competing interests.

## Authors’ contributions

CDR participated in the design of the study, carried out the genetic studies and statistical analysis and drafted the manuscript. MMH participated in the design of the study, in sample collection, helped carry out the genetic studies and helped to draft the manuscript. MRP participated in the design of the study and its coordination, and helped to draft the manuscript. JMN participated in the design of the study and its coordination and in sample collection, and helped to draft the manuscript. HGP participated in the design of the study and its coordination, and helped to draft the manuscript. AMB conceived of the study, and participated in its design and its coordination, helped carry out the genetic studies and helped to draft the manuscript. All authors read and approved the final manuscript.
